# Disruption in accessing sexual and reproductive health services among border populations during COVID-19 lockdown in Uganda

**DOI:** 10.7189/jogh.12.04065

**Published:** 2022-08-17

**Authors:** Paul Bukuluki, Peter Kisaakye, Francis Mulekya, John Mushomi, Chrispus Mayora, George Palattiyil, Dina Sidhva, Harish Nair

**Affiliations:** 1 Department of Social Work and Social Administration, School of Social Sciences, Makerere University, Kampala, Uganda; 2 Department of Population Studies, School of Statistics and Planning, Makerere University, Kampala, Uganda; 3 Department of Social Work and Social Administration, School of Social Sciences, Makerere University, Kampala, Uganda; 4 Department of Population Studies, School of Statistics and Planning, Makerere University, Kampala, Uganda; 5 Department of Health Policy, Planning and Management, School of Public Health, Makerere University, Kampala, Uganda; 6 Department of Social Work, School of Social and Political Science, The University of Edinburgh, Edinburgh, UK; 7 Department of Social Work, School of Education and Social Sciences, University of the West of Scotland, Paisley, UK; 8 Centre for Global Health, Usher Institute, The University of Edinburgh, Edinburgh, UK

## Abstract

**Background:**

The spread of COVID-19 exposed the inadequacies inherent in the health care systems of many countries. COVID-19 and the attendant demands for emergency treatment and management put a significant strain on countries’ health care systems, including hitherto strong health systems. In Uganda, as the government strived to contain COVID-19, other essential health care services were either disrupted or completely crowded out. Balancing the provision of COVID-19 treatment and management services and at the same time offering sexual and reproductive health and rights services (SRHR) proved to be a considerable challenge in these circumstances. COVID-19 prevention-related travel restrictions and border closures had far-reaching negative consequences on the mobility of individuals to access essential health services in Uganda. The situation may have been worse for cross-border communities that sometimes access services across the borders.

**Methods:**

Using quantitative data from 1521 respondents and qualitative data (20 key informant interviews and 12 focus group discussions), we investigate the disruption in accessing SRHR services for border communities in Uganda during COVID-19.

**Results:**

Results indicate that females (adjusted odds ratio (aOR) = 1.3; 95% confidence interval CI = 1.08-1.79), those with primary education (aOR = 1.47; 95% CI = 1.61-2.57), currently employed (aOR = 2.03; 95% CI = 1.61-2.57) and those with the intention to leave current residence (aOR = 2.09; 95% CI = 1.23-3.55) were more likely to have experienced a disruption in accessing SRHR services. However, respondents aged 35 years, or more were less likely to have experienced a disruption compared to their younger counterparts.

**Conclusions:**

Results shed light on the disruption of access toSRHR services during pandemics such as COVID-19 among a highly mobile population. There is a need to invest in building strong and resilient health care systems that can guarantee continuous access to essential health services including SRHR provisions among mobile populations during pandemics.

The novel coronavirus which was first detected in the Hubei province of China in November-December 2019 [1] quickly spread across the world, threatening global health security [2]. The World Health Organization (WHO) declared COVID-19 a global pandemic and called for a strong response from countries [3]. In response, countries across the world swiftly instituted various preventive and mitigation measures that were both country-context specific but also those that had been globally recommended and proven to be effective in other settings. The COVID-19 preventive measures largely included lockdowns, international travel restrictions, masking, and social distancing, among others. These measures had varied effects – intended and unintended – on various sections of the population. In Uganda, for example, during the intensive stage of lockdown, there were reports of missed childhood immunization schedules, increased home deliveries and some reported deaths of pregnant women enroute to health facilities [4-6]. This was largely due to travel restrictions, high transport costs, as well as measures put in place to decongest health centres [7,8]. Previous research has reported that access to sexual and reproductive health and rights services (SRHR) services is problematic among border communities or among populations on the move [9-11]. Yet, increasing access to SRHR in times of a pandemic is crucial to promoting healthy lives and well-being for all as is enshrined in Goal 3 of the Sustainable Development Goals (SDGs) [12,13]. The Global Compact on Migration (2018) also calls for the Member States to promote the health of migrants and host communities [14]. Some reports indicated that as the government focused on containing COVID-19, focus and resources were turned away from and crowded out other essential health services [15]. As a result, people have experienced poor SRHR outcomes due to limited access to SRHR services during COVID-19 [16-18].

Uganda is surrounded by its neighbours Democratic Republic of Congo (DRC), Kenya, Rwanda, Tanzania, and South Sudan – countries whose communities have historical links and connections were only separated by artificial colonial borders. It is not uncommon to find the same community split across and residing in two countries while being socially and economically interdependent [19]. Border communities often cross the borders for one reason or another, including accessing health services in a neighbouring country [20-22].

Uganda instituted cross-border movement restrictions to prevent the spread of COVID-19. Accordingly, all Ports of Entry (PoE) that are formally managed and supervised by the Uganda Immigration Authority were closed. There was also heightened security deployment at the porous borders and multiple points of entry that the immigration department was not in-charge or even not aware of. These disruptions in normal life for border communities led to reduced access to essential health care including SRHR services.

Therefore, this study sought to assess the disruption in accessing SRHR services among border populations. Respondents were asked whether they have ever experienced any disruption in accessing SRHR services during COVID-19. Those who experienced a disruption in accessing SRHR services during the COVID-19 lockdown were coded ‘Yes’, otherwise ‘No’.

## METHODS

### Study design and setting

The study employed an exploratory a mixed methods cross-sectional study design. We targeted individuals who were 18 years or older and living in the selected border communities or study areas.

This study was conducted in two border communities – Mutukula (Kyotera district) at the Uganda-Tanzania border, and Busia (Busia district) at the Uganda-Kenya border. These two points of entry (POE) were purposively selected because of the intense cross-border activity which happens there and has often involved formal and informal cross-border movements for members of communities who are resident in those places. Furthermore, the two points of entry have communities that share historical linkages across the border and these historical connections are partly associated with the cross-border movements within these communities. It should also be noted that the populations from Kenya and Uganda on Busia and Mutukula borders share health facilities.

### Sampling, and sample size

Using the Cochran formula for estimating samples for the quantitative strand [23], a sample size of 1793 was calculated assuming a response rate of 85%. A two-stage cluster sampling methodology was used to select 1521 respondents. In the first stage 12 clusters (villages) were sampled for each of the two communities. In the second stage, 75 households were randomly sampled from each cluster. A random number table was used to select the first household that acted as the reference point for the next household in front of its main door. It is at this next household that the first questionnaire was administered. The next household front door was selected until all the households needed in a chosen area were interviewed. The household head (usually the father) or the immediate available responsible member of the household above the age of 18 was interviewed.

### Data collection and ethical clearance

Data collection took place between July 17, 2021 and August 10, 2021, immediately after the pre-test (which took place on the July 15, 2021) – and the information from the pre-test guided the fine-tuning of the final questionnaire. The quantitative survey tool was programmed using Computer Assisted Personal Interviewing approach and captured on tablets using Kobo collect software. After quantitative data collection, data were downloaded from the server into the STATA software version 15 [24].

Qualitative data were collected by well-trained research assistants (3 female and 3 male) – who were graduate students. We used purposive sampling technique to select respondents for qualitative data. We conducted face-to-face interviews, and in some instances telephone interviews (especially for busy respondents who could not be available for physical interviews). The sample size was determined on the principle of topical saturation [25]. The respondents for the qualitative strand included individuals deemed to be central to enforcing and implementing COVID-19 preventive measures, including village health teams (VHTs), area local leaders, district health officials, migration officers, religious leaders, and civil society organisations (CSOs), as well as any implementing partners involved in the COVID-19 response framework within the selected areas. A pre-tested interview guide was used to conduct 20 key informant interviews (KIIs) in total – 10 in each study district and 12 focus group discussions (FGDs) – 6 in each study district. The categories for the FGDs included clearing and forwarding agents (female and male), female commercial sex workers, female traders, female youth (15-24 years), male traders and male truck drivers. On average, KIIs lasted for 1 hour and 30 minutes while FGDs lasted for 2 hours and 30 minutes. Interviews were conducted from the respondent’s place of work or household – with no one present during the interview besides the participants and researchers.

Atlas.ti was used to analyse qualitative data [26,27]. Qualitative interviews were audio-recorded and later transcribed verbatim and cleaned before use. Three coders coded the data under the supervision of an experienced researcher. The transcripts were complemented with field notes that were written by the FGD moderator and the KII interviewers during the interview session. Transcripts were not returned to participants to ensure correctness, but the research assistants were supervised by the study team, who double checked all data collected daily to ensure completeness and quality control.

Ethical clearance to conduct the study was granted by the Mildmay Uganda Research and Ethics Committee (MUREC) – REC 0706-2021. The ethical approval included all aspects of both the quantitative and qualitative methodology of the study. Respondents were assured of their safety, privacy, and confidentiality. Respondents were assured that they would remain anonymous and voluntary participation was central to study participation. We obtained verbal informed consent from respondents at the start of the interview. Respondents were made to understand that by agreeing to be interviewed, they were consenting to be part of the study. Verbal informed consent reduced the risk of transmitting COVID-19 through handling of paper and pen. Identifiers instead of participant names were used to ensure confidentially. Data were kept under strict security and only accessible to the study team, for purposes of this study only. Research findings from this study were analysed and reported in aggregate form. We adhered to the principles of Do-No-Harm when collecting data during public emergencies [28].

### Dependent variable

Respondents were asked whether they ever experienced a disruption in accessing SRHR services (Have you ever experienced any disruption in accessing SRHR services during COVID-19?). Those who experienced a disruption in accessing SRHR services during the COVID-19 lockdown were coded ‘Yes’, otherwise ‘No’. The outcome variable is binary because the responses are either ‘Yes’ or ‘No’.

### Independent variables

Background factors included sex (female or male), age (categorized in 10-year age groups and one open age group (65+). Respondents were asked their current marital status (either currently married or not). Level of education had four categories (no education, primary, secondary, or tertiary). We collected information on current employment status (currently employed or not). Respondents were asked whether they changed residence during COVID-19 or had an intention to leave their current residence. A response to each of the questions was either ‘Yes’ or ‘No’.

We collected information on respondents’ perceptions regarding COVID-19 transmission. That is, respondents were asked whether COVID-19 can be transmitted through coughing, not wearing a face mask, not keeping physical or social distance and whether they perceived themselves as being at risk of contracting COVID-19. A response to each of the questions was either ‘Yes’ or ‘No’.

### Data analysis

Analysis of the quantitative data was done using STATA software version 15 [24]. Frequency distributions and Pearson χ^2^ test was used to test the association between selected variables and disruption in accessing SRHR services during COVID-19 lockdown. We fitted a binary logistic regression model (because the outcome variable is binary) to identify the factors that were associated with the disruption in accessing SRHR services during the COVID-19 lockdown. We ran three models: Model 1 controlling for background factors; model 2 controlling for perception factors; and model 3 controlling for all factors.

Deductive coding was used to analyse qualitative data and integrated into the analysis of quantitative results [29]. Qualitative data were first transcribed from the local languages to English. Codes were then generated in relation to the selected factors included in the study – which can be used to explain a disruption in accessing SRHR services during COVID-19 lockdown. Quotes that relate to themes and sub-themes were extracted and included in the results. The triangulation of qualitative and quantitative results assisted in elucidating the findings.

## RESULTS

### Distribution of respondents

**Table 1** shows the distribution of respondents’ characteristics. Most respondents were male (61%), currently married (76%) and currently employed (62%). Slightly more than 90% did not change residence or had no intention of leaving their current residence during COVID-19. The mean age of respondents was 43 years. Less than half (46%) had primary education – with the least proportion of respondents with tertiary education (9%).

**Table 1:** Distribution of respondents by background factors

Background factors	Category	Frequency (n)	Percent (%)
Sex	Female	591	38.9
	Male	930	61.1
Age (mean age = 43 y; SD = 15 y)	15-24	120	7.9
	25-34	391	25.7
	35-44	359	23.6
	45-54	303	19.0
	55-64	198	13.0
	65+	150	9.9
Current marital status	Currently married	1154	75.9
	Not currently married	367	24.1
Level of education	No education	326	21.4
	Primary	694	45.6
	Secondary	371	24.4
	Tertiary	130	8.6
Currently employed	No	585	38.5
	Yes	936	61.5
Changed residence during COVID-19	No	1433	94.2
	Yes	88	5.8
Intention to leave current residence	No	1384	91.0
	Yes	137	9.0
Total		1521	100

**Table 2** shows results related to respondents’ myths, perceptions of risks and vulnerability to COVID-19. Nearly all respondents interviewed had a positive perception towards COVID-19 transmission. That is, most respondents agreed that COVID-19 can be transmitted through coughing (99%), not wearing a face mask (95%), and not keeping a social distance (98%). About 8 respondents out of every 10 perceived themselves as being at risk of contracting COVID-19.

**Table 2:** Myths, perceptions of risk and vulnerability to COVID-19

Variable	Category	Frequency (n)	Percent (%)
Whether COVID-19 can be transmitted through coughing	No	12	0.8
	Yes	1509	99.2
Whether wearing a facemask protects one from contracting COVID-19	No	81	5.3
	Yes	1440	94.7
Whether social distancing prevents contracting COVID-19	No	30	2.0
	Yes	1491	98.0
Whether perceive yourself to being at risk of contracting COVID-19	No	220	14.5
	Yes	1301	85.5
Total		1521	100

### Relationship between selected background factors and disruption in reproductive health services

**Table 3** shows that respondents' access to SRHR services was significantly different by sex (*P* < 0.05), age (*P* < 0.01), current marital status (*P* < 0.05), level of education (*P* < 0.05), current employment status (*P* < 0.001), intention to leave current residence (*P* < 0.01) and perceptions regarding risks of contracting COVID-19 *P* < 0.001). Overall, the results in **Table 3** indicate that majority of respondents (72%) experienced a disruption in accessing SRHR services. Female respondents who experienced a disruption in accessing SRHR differed from those who did not by about 46% while male respondents who experienced a disruption in accessing SRHR differed from those who did not by approximately a third (34%).

**Table 3:** Relationship between selected factors and disruption in accessing sexual and reproductive health services during COVID-19 lockdown

Variable	Category	Disruption in accessing reproductive health services, % (No.)		χ^2^ (*P*-value)
		**No**	**Yes**	
Sex	Female	27.2 (161)	72.8 (430)	5.0 (0.025)
	Male	32.7 (304)	67.3 (626)	
Age	15-24	19.2 (23)	80.8 (97)	21.7 (0.001)
	25-34	24.6 (96)	75.4 (295)	
	35-44	35.9 (129)	64.1 (230)	
	45-54	34.7 (105	65.3 (198)	
	55-64	31.8 (63)	68.2 (135)	
	65+	32.7 (49)	67.3 (101)	
Current marital status	Currently married	31.9 (368)	68.1 (786)	3.9 (0.048)
	Not currently married	26.4 (97)	73.6 (270)	
Level of education	No education	35.6 (116)	64.4 (210)	10.8 (0.013)
	Primary	27.4 (190)	72.6 (504)	
	Secondary	33.9 (126)	66.0 (245)	
	Tertiary	25.4 (33)	74.6 (97)	
Currently employed	No	38.8 (227)	61.2 (358)	30.3 (<0.001)
	Yes	25.4 (238)	74.6 (698)	
Changed residence during COVID-19	No	30.8 (442)	69.2 (991)	0.9 (0.352)
	Yes	26.1 (23)	73.9 (65)	
Intention to leave current residence	No	31.9 (441)	68.1 (943)	12.1 (0.001)
	Yes	17.5 (24)	82.5 (113)	
Whether COVID-19 can be transmitted through coughing	No	25.0 (3)	75.0 (9)	0.2 (0.674)
	Yes	30.6 (462)	69.4 (1047)	
Whether wearing a facemask protects one from contracting COVID-19	No	24.7 (20)	75.3 (61)	1.4 (0.238)
	Yes	30.9 (445)	69.1 (995)	
Whether social distancing prevents contracting COVID-19	No	20.0 (6)	80.0 (24)	1.6 (0.204)
	Yes	30.8 (459)	69.2 (1032)	
Whether perceive yourself to being at risk of contracting COVID-19	No	44.1 (97)	55.9 (123)	22.1 (<0.001)
	Yes	28.3 (368)	71.7 (933)	
Total		465 (30.6)	1056 (69.4)	

Results from the qualitative interviews and FGDs also point to nuances that reflect experiences of disruption of access to SRHR services due to COVID-19 and its control measures. Some participants especially health workers noted that a phobia of being susceptible to the risk of contracting COVID-19 discouraged them from providing SRHR services at the facility and through community outreaches. This is more so because several health workers contracted COVID-19 during the process of rendering services to the population.

*Fear to contract COVID-19 also stopped some of us from extending SRHR services because one of our staff got infected and she was off-duty for about 2 weeks, this scared a lot of staff and also the patients decided to shun the facility and any other community health programs that could be extending SRHR services,* (KII with In-charge at Mutukula Border Town).

The other explanation provided for the disruption of services by health providers was that COVID-19 and associated restrictions in movement interfered with access to SRHR services offered by facilities that were quite distant from the service users. To cope with this situation, some of the users opted to seek services from traditional birth attendants or lay health workers. Similarly, some potential service users of SRHR services were not comfortable with wearing face masks because they perceived them as an inconvenience and therefore opted to stay at home.

*We didn’t perform so well on delivery. People preferred to go to the TBAs (Traditional Birth Attendants) who were near them than coming to the facility. They had stigma that when you go to the facility, they will test you and tell you that you have COVID-19 and they lock you in your house for 14 days so they feared to come* (KII with the In-charge Buteba Health Centre III).

*People did not want to wear facemasks and because of this measure, many opted to stay at home because they thought wearing facemasks was disturbing. Schools were closed and there was no way information could be passed to pupils and students because they were home. We couldn’t also do community outreach because gatherings were being banned and it was not easy to individually meet people* (KII with Nurse at Mutukula Border Town).

Furthermore, the effects of COVID-19 on people’s livelihoods and sources of income forced them to prioritize basic needs like food over seeking SRHR services. Quite a number of them noted that they could barely survive and therefore had to make some hard choices between seeking health care and survival.

*Poverty as many people were not working at the time, many were not even interested in going for those services as they needed to have some money but had no source of income. Services were available but since people were not working, they were not interested to go for them because they were poor* (IDI with VHT, Dabani Sub-County Busia District).

### Factors associated with disruption with accessing sexual and reproductive health services among populations in border districts during COVID-19 lockdown

**Table 4** shows results (from 3 models) of the factors that were significantly associated with access to SRHR services during COVID-19 lockdown. Model 1 controlled for only background factors. Model 2 controlled for myths, perceptions of risk and vulnerability to COVID-19 and the third model (model 3) controlled for all the factors. The results from model 3 indicate a similar pattern (in terms of magnitude and direction) to the results in models 1 and 2. Females (OR = 1.39; 95% CI = 1.08-1.79) were more likely than men to have experienced a disruption in SRHR services. Respondents aged 35-44 years (OR = 0.67; 95% CI = 0.21-0.64), 45-54 years (OR = 0.39; 95% CI = 0.22-0.68), 55-64 years (OR = 0.43; 95% CI = 0.24-0.79) and those aged 65 years or more (OR = 0.46; 95% CI = 0.25-0.85) were all less likely to have experienced a disruption in accessing SRHR services during COVID-19 lockdown than their counterparts aged 15-24 years.

**Table 4:** Factors associated with disruption in accessing sexual and reproductive health services among populations in border districts during COVID-19 lockdown*

Variable	Category	Model 1	Model 2	Model 3
Sex	(RC = Male)	1		1
	Female	1.38 (1.08-1.77)†		1.39 (1.08-1.79)‡
Age	(RC = 15-24)	1		1
	25-34	0.68 (0.39-1.15)		0.67 (0.39-1.15)
	35-44	0.38 (0.22-0.65)§		0.37 (0.21-0.64)§
	45-54	0.41 (0.24-0.70)‡		0.39 (0.22-0.68)‡
	55-64	0.48 (0.27-0.85)†		0.43 (0.24-0.79)‡
	65+	0.49 (0.27-0.89)†		0.46 (0.25-0.85)†
Current marital status	(RC = Currently married)	1		1
	Not currently married	1.18 (0.87-1.59)		1.18 (0.87-1.59)
Education	(RC = No education)	1		1
	Primary	1.47 (1.09-1.98)†		1.47 (1.61-2.57)†
	Secondary	0.91 (0.64-1.28)		0.90 (0.64-1.28)
	Tertiary	1.23 (0.76-1.99)		1.19 (0.73-1.95)
Currently employed	(RC = No)	1		1
	Yes	2.08 (1.65-2.63)§		2.03 (1.61-2.57)§
Changed residence during COVID-19	(RC = No)	1		1
	Yes	0.71 (0.39-1.26)		0.81 (0.45-1.46)
Intention to leave current residence	(RC = No)	1		1
	Yes	2.21 (1.30-3.74)‡		2.09 (1.23-3.55)‡
Whether COVID-19 can be transmitted through coughing	(RC = No)		1	1
	Yes		1.00 (0.23-4.41)	0.85 (0.19-3.79)
Whether wearing a facemask protects one from contracting COVID-19	(RC = No)		1	1
	Yes		0.76 (0.44-1.30)	0.76 (0.43-1.31)
Whether social distancing prevents contracting COVID-19	(RC = No)		1	1
	Yes		0.47 (0.17-1.30)	0.61 (0.22-1.67)
Whether perceive yourself to being at risk of contracting COVID-19	(RC = No)		1	1
	Yes		2.09 (1.55-2.81)§	2.10 (1.54-2.87)§
Constant		2.05 (1.15-3.66)†	3.32 (0.84-13.09)	2.85 (0.61-13.32)

RC – reference category

*Odds ratios and 95% confidence intervals for each model.

†*P* < 0.05.

‡*P* < 0.01.

§*P* < 0.001.

Respondents with primary education were more likely (OR = 1.47; 95% CI = 1.61-2.57) than their counterparts with no education to have experienced a disruption in accessing SRHR services during the COVID-19 lockdown. Currently employed respondents were more likely (OR = 2.03; 95% CI = 1.61-2.57) than respondents who were not currently employed to have experienced a disruption in accessing SRHR services during the COVID-19 lockdown. This finding may point to the fact that respondents with no education or the unemployed may either not be aware of SRHR services or may not be utilizing health services.

Those intending to leave the current residence were more likely (OR = 2.09; 95% CI = 1.23-3.55) to have experienced a disruption in accessing SRHR services during COVID-19 lockdown than respondents who were intending to remain at the current residence. The results in **Table 4** indicate that respondents that perceived themselves being as at risk of contracting COVID-19 were more likely (OR = 2.10; 95% CI = 1.54-2.87) to have experienced a disruption in accessing SRHR services during COVID-19 lockdown that their counterparts who did not perceive themselves being at risk.

Similar to quantitative findings, several participants noted that travel to health facilities due to COVID-19 lockdown including harassment and mistreatment from security agencies and providing detailed explanations and documented evidence to be allowed to go to health facilities disrupted their access to SRHR services.

*The army men were rude to people. They were beating people, and this was scaring them to access the facilities. As for the out reaches, we were not going beyond the home area because we were also scared like VHTs and we were not recognized as health workers* (KII with VHT, Dabani Sub-County Busia District).

Some of the participants noted that they could not access essential commodities and supplies for prevention of STIs, HIV and pregnancy due to the lockdown and its associated restrictions. Similarly, some mothers due to give birth opted to deliver at home or under the care of traditional birth attendants. Some of the mothers who had high-risk pregnancies and would ordinarily seek to deliver at tertiary level or regional referral hospitals decided to deliver at nearby primary health care facilities because they found it difficult to navigate access to these higher level. Several participants also noted that they missed antenatal care (ANC visits) and could not attend postnatal care as required due to the high costs of hiring private transport means and the need to get movement permits especially during the tight lockdowns.

*STDs, STIs and HIV/AIDS contracting cases increased because people had no protective measures and even the condoms that were available expired since no one could reach the health facilities to access them. On delivery care, many pregnant mothers opted for traditional methods of delivery since accessing health facilities was a big problem and the transport costs were too high. Medical supplies also went down because the numbers were few and the ministry could not supply the services when there is no one to access them* (KII with one of the Health Inspectors at Mutukula border District).

*Since people were at home, many did not take family planning as a priority. With access to condoms, some people failed to access them because they couldn’t reach the health facilities and we couldn’t also access the VHTs in order to extend or make sure that these materials reach communities. Generally, the levels of access to SHR services reduced during the lockdown*, (KII, Health worker at Mutukula border District).

*The numbers of mothers who would come for antenatal care services because challenges in using their usual means of transport, when it comes to family planning. Many mothers who would come to deliver from the hospital ended up actually delivering from nearby health center III’s despite the fact that some of them by the nature of the risk they had, they were not meant to deliver from those health centers* (KII, with doctor, Dabani hospital, Busia).

*Since people could not move freely the turn up to access services as they could not explain themselves to the law enforcement officers that they were going to health facilities to pick condoms because of restrictions on movements. The Ministry of Health became reluctant in sending these services since the turn up was low. Curfew also affected access to SHR services since people were not moving freely and the turn up of the youth to access these services came down and so did the supplies since supplies depend on the number of clients you receive as a facility,* (KII Health official at Mutukula border District).

*Others were coming late, some were unable to come for antenatal care, few mothers coming for antenatal care, family planning services, postnatal care. Consequently, we had mothers with unfavorable outcomes from labor, as babies were coming out very tired and some of them were already dead. You would get a mother who was referred early but because of the process like transport, authorization letters, you find that the mother is coming but the baby is dead. Generally, there was a lot of delay, an increase in what we call perinatal death. The babies died around that period before delivery, after delivery immediately and very sick newborn*, (KII with health worker, Hospital Busia Border District).

There were also challenges shared by health workers that affected their capacity to provide SRHR services during the COVID-19 lockdowns. These ranged from inability to travel to places of work or health facilities due to high transport costs, reduced supplies and delayed deliveries of medicines, equipment and other health supplies leading to stock-outs.

*Some health workers were not working due to the high transport costs. We also had limited supplies for example, the ministry reduced the amount of medical equipment during the lockdown due to the low demand as people were no longer coming at the facility. Health workers had negative attitudes towards clients because they also had the fear of contracting COVID-19 from these patients*, (KII Health inspector Mutukula border District).

*The challenge was the stock out, NMS delayed delivering drugs, medicines and supplies during that lockdown. They took a very long time and I remember that time, we received even two cycles instead of one because of the delay*, (KII Buteba Health Centre III Busia).

## DISCUSSION

We investigated the disruption in accessing SRHR services in Uganda during COVID-19 among populations in Busia and Mutukula border districts in East and Southwestern Uganda respectively. Evidence from this study can inform policy, preparedness, and programming to overcome access barriers to SRHR services during pandemics such as COVID-19 among mobile populations. The findings can help inform the formulations of effective mechanisms for engagement of cross-border populations in joint risk analysis and developing actions to increase access to health facilities for SRHR services in times of a pandemic [30]. This study shows that most respondents in border areas experienced disruptions in access to SRHR services due to COVID-19 and its control measures such as lockdowns and restrictions in movement. It reaffirms findings from related studies showing that COVID-19 disrupted access to sexual and reproductive health services for mobile populations including those at border points or areas [9,31-34]. This implies that guaranteeing the SRHR of populations living in fragile settings including cross border settings like Mutukula and Busia amidst border closures and other movement restrictions similar to those experienced during lockdowns is essential and constitutes a basic human right [9,31,35].

The study reveals that the disruption of access to SRHR services is not equal among genders in border populations and requires further attention. For example, females were more likely than males to have experienced a disruption in SRHR services and qualitative data reveal that mothers due to give birth had limited options. These women opted to deliver from home or under the care of traditional birth attendants which potentially exposed them to higher risks. In addition, some inherently high-risk women were unable to deliver in centres with more skilled and specialized care. Our study also demonstrates disruptions in access to antenatal and postnatal care for expectant mothers due to COVID-19 and its control measures [8,35] including lockdowns, border closures limiting crossing the border to seek services from the nearest hospitals across the border, skyrocketing of transport costs due to banning of public transport and difficulties in accessing movement or travel permits during lockdowns [8,13,31,33]. Our results are similar to those of Church et al. [8] who observed that restrictions in movement coupled with reduction or suspension of mobile outreach programmes offering SRHR services heavily restricted women and girls from accessing essential SRHR services.

This study further affirms results from other studies that found the loss of livelihoods by those employed especially in the informal sector and private sector had disrupted access to SRHR services during COVID-19 [33,36,37]. In this respect qualitative results show that people tended to prioritize survival and meeting basic needs especially food and shelter over seeking SRHR services. This is similar to findings of other studies conducted in low-income settings [8,13,31,35].

Our results further demonstrate the link between migration/mobility intentions and how inability to travel or migrate due to COVID-19 movement restrictions contributed to disruption in access to SRHR services among migrants or people with intention to leave. Results shed light on the disruption of access to SRHR services during pandemics such as COVID-19 among a mobile population [7,9].

Our results show the role of intersectionality or intersections between age, gender, mobility, and access to SRHR during global pandemics particularly in border areas in low-income settings like Uganda. Apart from sex, age as a determining factor for disruption of access to SRHR services during COVID-19 in border areas with young people below 35 years more susceptible to experience a disruption compared to the older age groups. This demonstrates the urgency to prioritize the needs of adolescents and young people in cross-border areas during global pandemics similar to COVID-19 in low-income settings [13]. In addition, results from our study reveal the need to explore other intersections related to poverty, disability, ethnicity or race and migration and how these may influence access to SRHR during pandemic emergencies like COVID-19 [38]. Intersectionality facilitates shifting away from narrow focus on for example gender alone to broadening the understanding of the role of social determinants that combine with gender in influencing access to SRHR services in different contexts during pandemic emergencies such as the COVID-19 pandemic [38].

This study shows that respondents who perceived themselves as being at risk of contracting COVID-19 were more likely to have experienced a disruption in accessing SRHR services. This may point to the need for using integrated service delivery models that integrate SRHR and COVID-19 prevention and treatment messages in the context of managing and responding to pandemics [8,13].

Phobia for being susceptible to the risk of contracting COVID-19 during travel and while at service delivery points discourages potential service users from seeking SRHR services. Additionally, a phobia can make health workers become susceptible to stigmatization as potential transmitters of COVID-19 to their families [37]. This is more so because several health workers contracted COVID-19 during the process of rendering services to the population. This disrupted both the supply side of SRHR services as well as the demand for SRHR services. Other supply-related disruptions of access included the inability of health providers to travel to workplaces due to high transport costs, reduced supplies of medicines and equipment to delayed deliveries of medicines and health supplies leading to stockouts [8,13,33]. Supply side disruptions have been attributed in several contexts especially in lower-resourced countries particularly to funding for ongoing reproductive health programs being diverted to public health emergencies like the COVID-19 pandemic [32].

However, the results of this study should be treated with caution because of the following limitations. First, due to stigma, some respondents were reluctant to disclose their mobility patterns, nationality, and history of health conditions. This may lead to under-reporting of some indicators. Second, the results reported in the study may be affected by social-desirability bias given the sensitivity of questions and context of COVID-19. That is, some people may choose to provide answers they wish to be heard, especially to questions related to SRHR and perceptions about COVID-19 transmission.

## CONCLUSIONS

Participant experiences and the observations from interviews point to an experience of disrupted access to sexual and reproductive health services for border points or areas particularly affecting the women, young age groups, and those who had intentions to travel or migrate (mobility) and those who were employed and had disruptions in livelihoods due to COVID-19 related restrictions. While responding to future health emergencies and similar pandemics such as COVID-19, governments should remain alert so as not to drop the ball regarding the gains already made in other equally vital health matters such as SRHR and related issues. Further, the findings from this study demonstrate the need to invest in building a strong and resilient health care system and infrastructure that can guarantee continuous access to essential health services including SRHR services among mobile populations and borders during pandemics. They also point to the need for cross-border collaboration and integrated delivery of COVID-19 prevention services with other essential services, particularly SRHR. Given that the most affected population groups were women and young people, programming for SRHR during a pandemic should pay special attention to gendered dynamics that disrupt access to SRHR services during pandemics including power relations and access to and control over resources. Attention should also be focused on facilitating young age groups of adolescents and youth to address factors that increase disruption of access to SRHR services among these age groups during pandemics such as COVID-19 in border settings.
